# Physico-mechanical analysis data in support of compatibility of chitosan/κ-carrageenan polyelectrolyte films achieved by ascorbic acid, and the thermal degradation theory of κ-carrageenan influencing the properties of its blends

**DOI:** 10.1016/j.dib.2016.09.039

**Published:** 2016-10-01

**Authors:** Mahdiyar Shahbazi, Rammile Ettelaie, Ghadir Rajabzadeh

**Affiliations:** a Research Institute of Food Science and Technology (RIFST), Mashhad, Iran; b Food Colloids Group, School of Food Science and Nutrition, University of Leeds, Leeds LS2 9JT, United Kingdom

**Keywords:** Polyelectrolyte compatibility, Aggregation-precipitation complex, Mechanical properties, Surface hydrophobicity, Glycosidic bond degradation

## Abstract

This article presents the complementary data regarding compatibilization of chitosan/κ-carrageenan polyelectrolyte complex for synthesizing of a soft film using ascorbic acid. It includes the thermal-theory for estimating the degradation of κ-carrageenan, as reflected in alteration of the structural properties of the blend. The data has been provided to demonstrate that the blend solution based on chitosan, a polycation, and κ-carrageenan, a polyanion polymer, produces an incompatible polyelectrolyte composite, susceptible to coaservative phase separation. We present further data on water resistance, water barrier property, mechanical parameters, scanning electron micrograph, as well as contact angle image dataset of the chitosan/κ-carrageenan film. The physical data were collected by water solubility and water permeability assays, with a view to elucidate the role of ascorbic acid in the compatibility of polyelectrolyte blends. The mechanical data is obtained from a stress–strain curve for evaluation of tensile strength and elongation at break point of the chitosan/κ-carrageenan film. The microstructure observations were performed using scanning electron micrograph. These dataset confirm fabrication of a soft film in the presence of ascorbic acid, with reduced heterogeneities in the polyelectrolyte film structure. The κ-carrageenan was also treated by a thermal process, prior to inclusion into the chitosan solution, to investigate the impact of this on the mechanical and structural features of the resulting blend. We present the required data and the theoretical analysis supporting the thermal chain degradation of a polymer and its effects on behavior of the film. Additional information, characterizing the hydrophobicity of the surface of the blend layers is obtained by measuring water contact angles using a contact anglemeter.

**Specifications Table**

**Table:** 

Subject area	*Rheological science, Composite materials*
More specific subject area	*Polymer composite film*
Type of data	*Table, graph, image*
How data was acquired	*Low affinity of κ-carrageenan for chitosan by adding ascorbic acid was recognized through water resistance assay. Change in water vapor permeability of blends as affected by acidified solutions was evaluated by measuring water diffusivity through chitosan/κ-carrageenan film. Mechanical behavior of polyelectrolyte films with a low affinity characterized by a texture analyzer system. Surface morphology of compatibilised polyelectrolyte film was verified from SEM micrographs. Sorption isotherm of blend films was successfully modeled using GAB, BET and Smith equations. Surface hydrophobicity of polyelectrolyte blend films was characterized by measuring water contact angle photographs using a drop-shape analyzer.*
Data format	*Raw, plotted, analyzed, modeled*
Experimental factors	*Acetic acid/Ascorbic acid ratio, Relative humidity, Heating time.*
Experimental features	*The neat chitosan film solution was made by dispersing 1 g chitosan in 2%* (v/v) *of the acetic/ascorbic acids aqueous solution to obtain a concentration of 2 g/dL. The solution was stirred at 60 °C for 20 min. Separately, for the preparation of the treated blend; the κ-carrageenan solutions were heated at various thermal times at 80* ^*o*^*C, and were then incorporated into the chitosan solution to obtain a treated blend film.*
Data source location	*34.1918°N, 48.3627°E, Iran*
Data accessibility	*Data are available with this article.*

**Value of the data**

• Data presents detailed description of how a cationic polymer in acidified solution can be made to have low affinity for κ-carrageenan, where otherwise strong aggregation-precipitation is observed.
• Physical and mechanical data are valuable to elucidate the role of ascorbic acid in compatibilisation of an incompatible blend.
• SEM photomicrograph dataset shows the possibility of producing a uniform polyelectrolyte films, with combination of acetic and ascorbic acid, from biopolymer blends that otherwise will result in highly non-uniform films.
• Data shows detailed description of degradation behavior of κ-carrageenan chains under long-time heating.
• Data provides equilibrium moisture content analysis of all polyelectrolyte films, ensuring the accuracy of the GAB model, as well as indicating usefulness of blends as moisture barrier.

## 1 Data

**Fig. 1:**
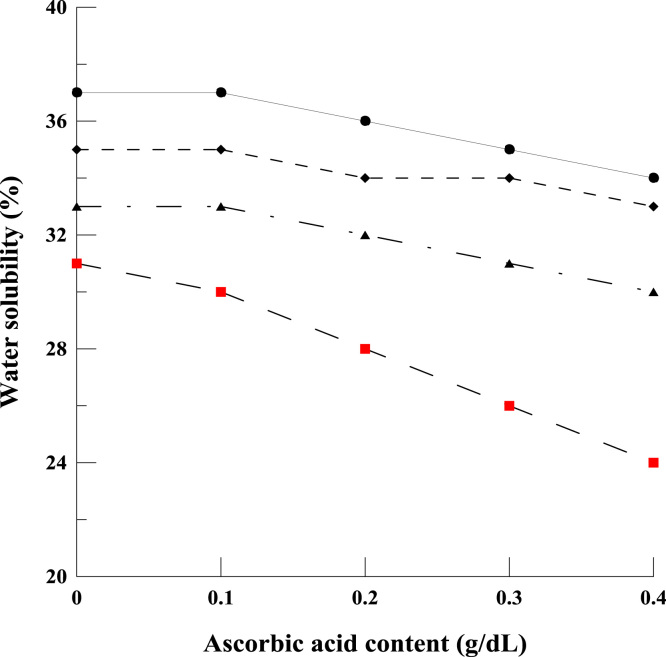
Water solubility of chitosan/κ-carrageenan film containing different levels of acidified solutions. The symbols are the blend consisting: 0.5 (•), 1 (♦), 1.5 (▲) and 2% (v/v) (■) of acetic acid.

**Fig. 2:**
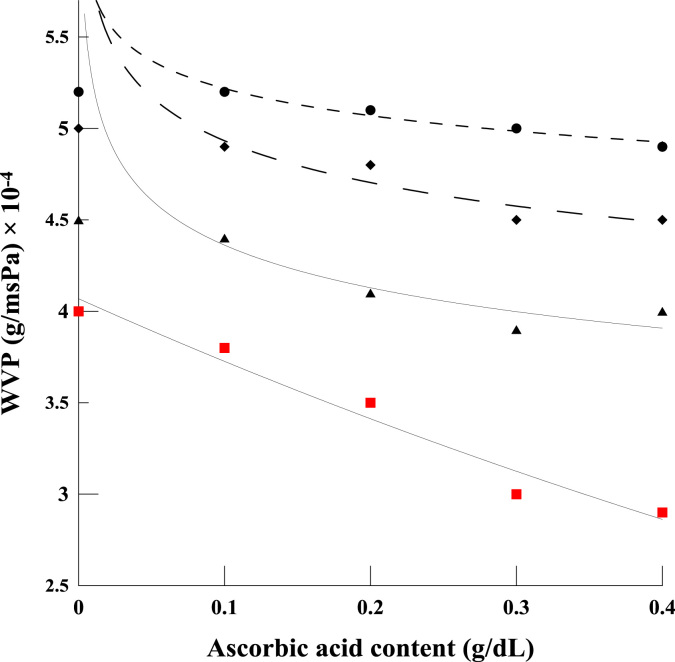
Change of water vapor permeability of chitosan/κ-carrageenan film plotted as a function of ascorbic acid concentration, for blends also containing various amount of acetic acid; 0.5 (•), 1 (♦), 1.5 (▲) and 2% (v/v) (■).

**Fig. 3:**
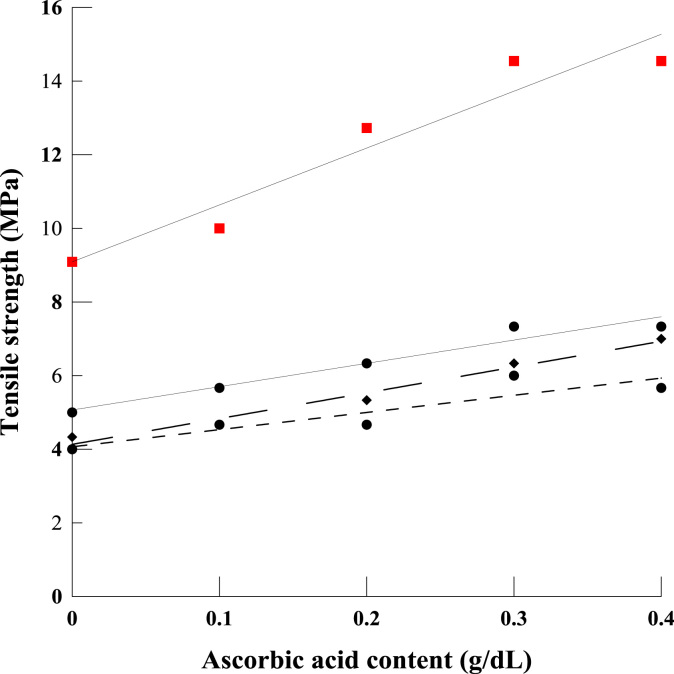
Tensile strength of the blend film measured in various contents of the acidified solution. The symbols indicate blends that also contain varying amounts of acetic acid as follows: 0.5 (•), 1 (♦), 1.5 (▲) and 2% (v/v) (■).

**Fig. 4:**
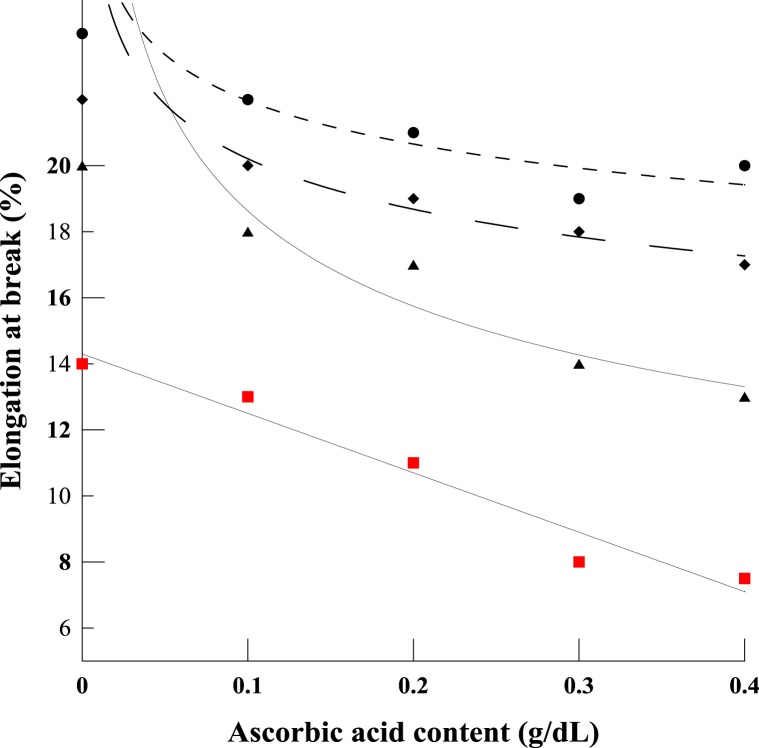
Elongation at the break point of the chitosan/κ-carrageenan film derived from the stress-stress curves obtained by texture analyzer and analyzed using it associated software. The symbols are the blends consisting of: 0.5 (•), 1 (♦), 1.5 (▲) and 2% (v/v) (■) of acetic acid, respectively.

**Fig. 5:**
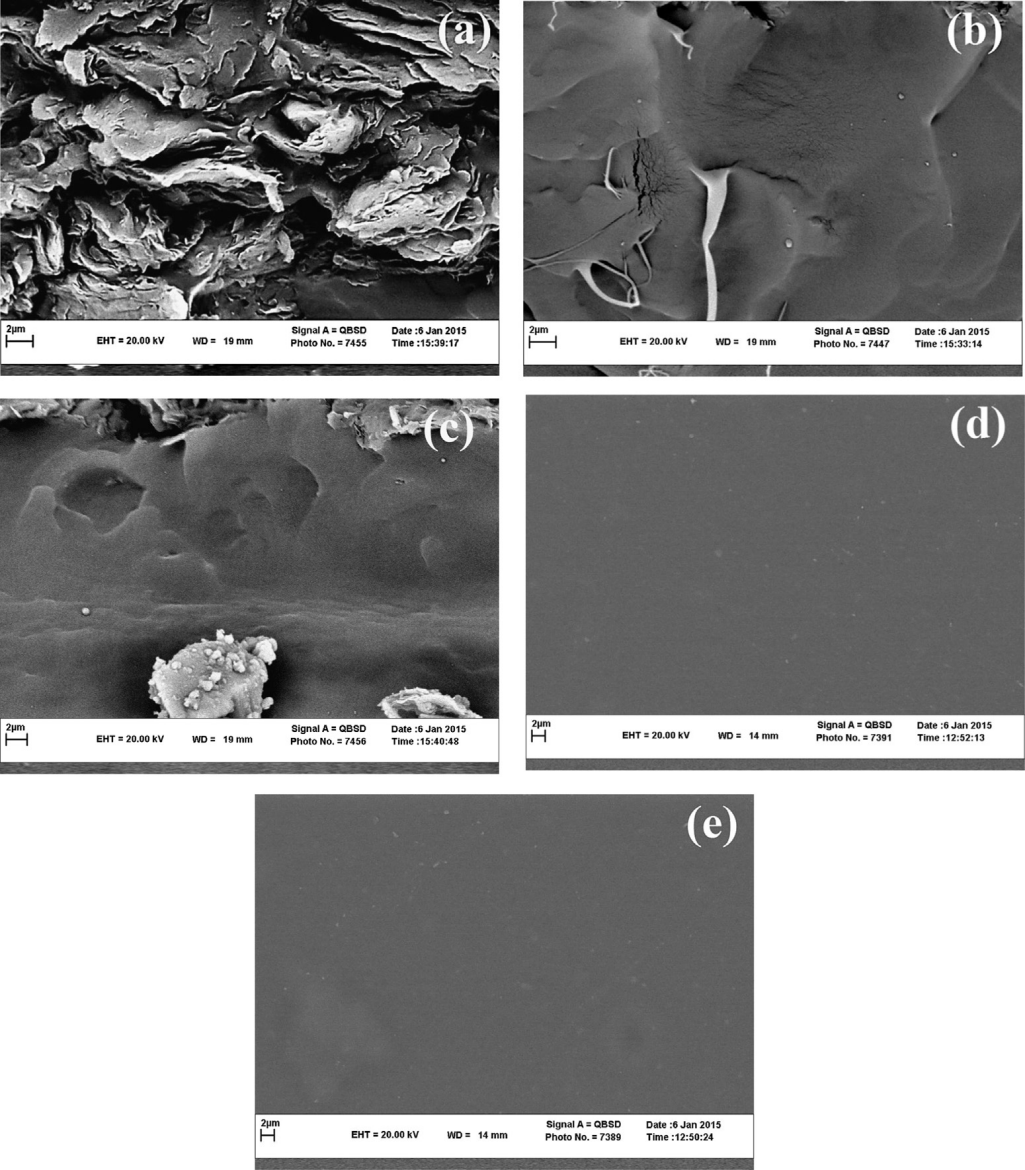
SEM photomicrographs of the blend film in the presence of acetic acid of 2% (v/v). 0 (a), 0.1 (b), 0.2 (c), 0.3 (d) and 0.4 g/dL (e) of the ascorbic acid. Magnification is 10.00 kX. Uniform structures for the case (c) and (d) are clearly evident here.

The shared data provide details on the fabrication of a compatible polyelectrolyte blend film based on chitosan and κ-carrageenan, using a combination of acetic and ascorbic acids. The data provide information on water solubility (Fig. 1), total soluble matter, water barrier property (Fig. 2) and mechanical properties of polyelectrolyte films (Fig. 3, Fig. 4). Further data on Morphological observation of the films, treated by the acidified solutions, are also presented in Fig. 5. The equation for the change in molecular weight of κ-carrageenan against thermal degradation time, based on a first order breakage of bonds, is derivate.

## 2 Experimental design, materials and methods

### 2.1 Materials

The chitosan/κ-carrageenan polyelectrolyte film was prepared as described in the associated research article [1]. The acetic acid and L-ascorbic acid were purchased from Sigma-Aldrich (St. Louis, MO, USA). All other chemicals were analytical reagent grade.

### 2.2 Film preparation

The κ-carrageenan that was previously prepared as described in [1], in its disordered state, was introduced into chitosan-based acidified solution. A binary solution based on acetic acid and ascorbic acid, as a crosslinker, was also used for preparing the chitosan/κ-carrageenan polyelectrolyte film. Various level of acetic acid (0, 0.5, 1.5 and 2% v/v) and ascorbic acid (0, 0.1, 0.2, 0.3 and 0.4 g/dL) was incorporated in the chitosan + κ-carrageenan blend solution. The blend films were poured into the glass container and transferred to an oven at 104 °C overnight in order to remove the residual solvent. Then, the dried film were peeled off from the glass container.

### 2.3 Statistical analysis

The measurement analysis of data on completely randomized design (CRD) was performed with the analysis of variance (ANOVA) followed by Duncan׳s test procedure in SPSS (Version 19, SPSS Inc., Chicago, IL). A value of *p*<0.05 was considered statistically significant. The measurements of all experiments were analyzed in triplicate and the mean and standard deviations of the data were reported.

### 2.4 Evaluation of compatible blend polyelectrolyte film

#### 2.4.1 Water resistance data

The data provided here is for water solubility of the chitosan/κ-carrageenan polyelectrolyte blend films with different contents of the acetic and ascorbic acids (Fig. 1). It is intended to show possible improvements in the dissolution resistance of the blend films arising from the addition of a combination of these two acids.

#### 2.4.2 Water barrier property data

The water vapor permeability (WVP) dataset are presented in Fig. 2. Change in WVP of the polyelectrolyte blend films as affected by acidified solutions was measured by gravimetric method following ASTM E398-03 [2]. Graphs show the results obtained for different amounts of acetic acid, ranging from 0.5% to 2%, with WVP plotted against the concentration of ascorbic acid.

#### 2.4.3 Mechanical characterization dataset

The mechanical parameters for the chitosan/κ-carrageenan polyelectrolyte blend films were inferred from stress/strain curves, obtained using a texture analyzer device (TA-XT_2_, UK). The tensile strength data was determined by stretching the specimen in order to establish a maximum stress. Elongation at break data was obtained by the ratio of the film elongation at the point of rupture to its initial length. As in the curves of Section 2.4.2, different graphs of Fig. 3 represent varying concentrations of acetic acid incorporated into the blends. The measured tensile strength is plotted against the concentration of ascorbic acid. In a similar way, graphs in Fig. 4 present associated data for the elongation of the films under the application of stress, as seen just prior to their final breakage. Taken together, the above two sets of data can be used to optimize the design of the blends for best strength vs elongation properties.

#### 2.4.4 Microstructure observations dataset

Scanning electron microscopy (SEM) experiment was intended to evaluate the compatibility and miscibility of the blend film based on chitosan and κ-carrageenan. The microstructure images here display a representative structure for the polyelectrolyte blend containing 2% (v/v) acetic acid, at the higher levels of ascorbic acid. The heterogeneous nature of the matrix with a non-uniform surface is evident for the film that contains low levels of ascorbic acid, as clearly observed in Fig. 5a–c. On the contrary, the matrix of the blend film became more uniform when ascorbic acid was incorporated into the system at levels of 0.3 and 0.4 g/dL (Fig. 5d–e).

### 2.5 Data relating to thermally treated blend films

**Scheme 1:**
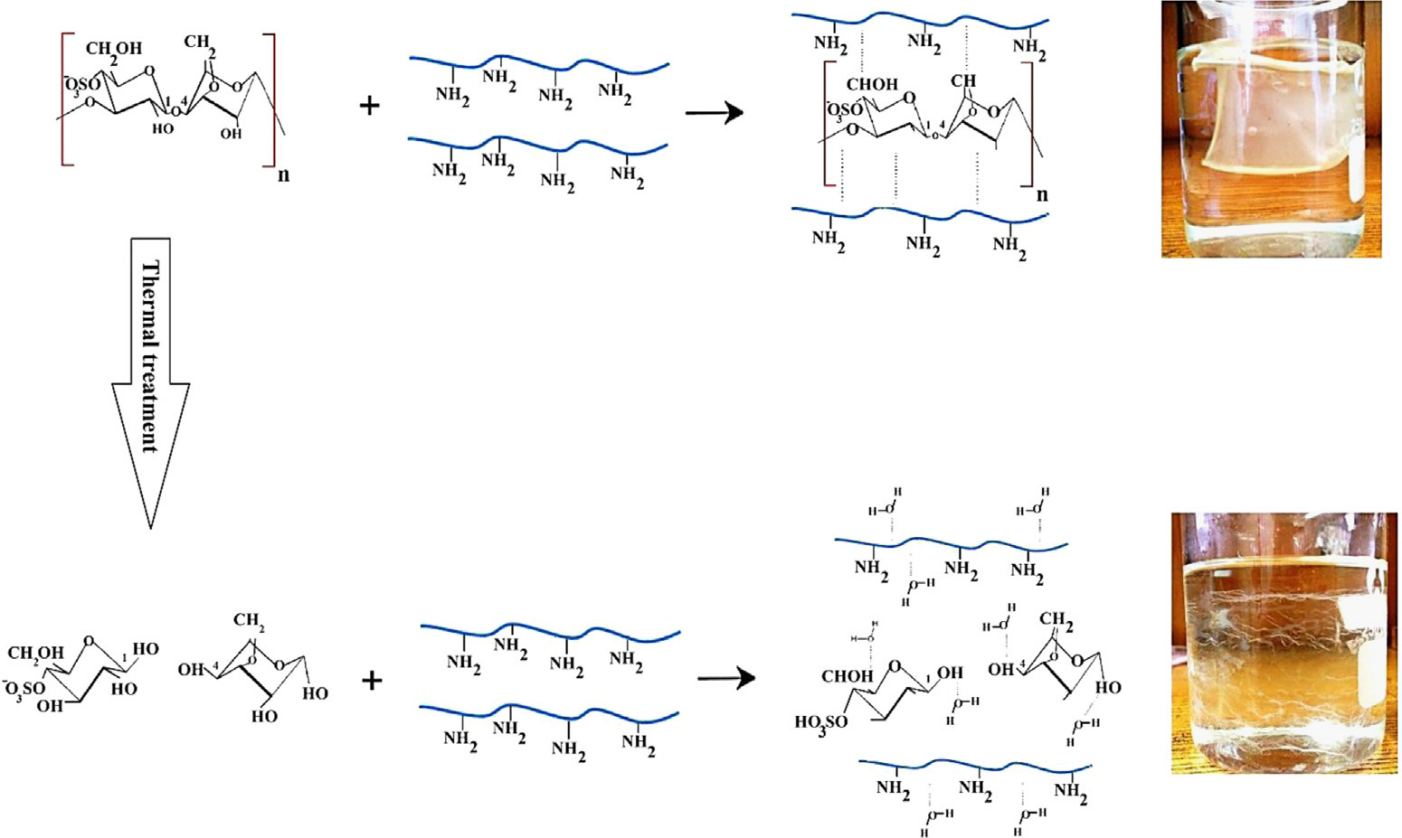
The chain scission of κ-carrageenan by thermal degradation of α (1→3) and β (1→4) glycoside bonds. Then, incorporation of the thermally treated and untreated κ-carrageenan into the chitosan solution for making the blend film. Graphical abstract adopted from Shahbazi, Rajabzadeh, Ettelaie and Rafe [1].

κ-carrageenan was also treated by a thermal process at various time durations, and then successfully introduced into the chitosan solution to evaluate the effect of the thermal conditioning on the physical and structural characteristics of the resulting blend film. More details are provided in our related reported work [1]. In the following sections, we present our derivation of the manner in which the molecular weight of the chains alters as a function of applied thermal time, based on secession of κ-carrageenan following a *pseudo*-first order kinetic during the period of the thermal treatment (Scheme 1).

#### 2.5.1 Derivation of degradation kinetics used in the analysis of thermal degradation data

**Scheme 2:**
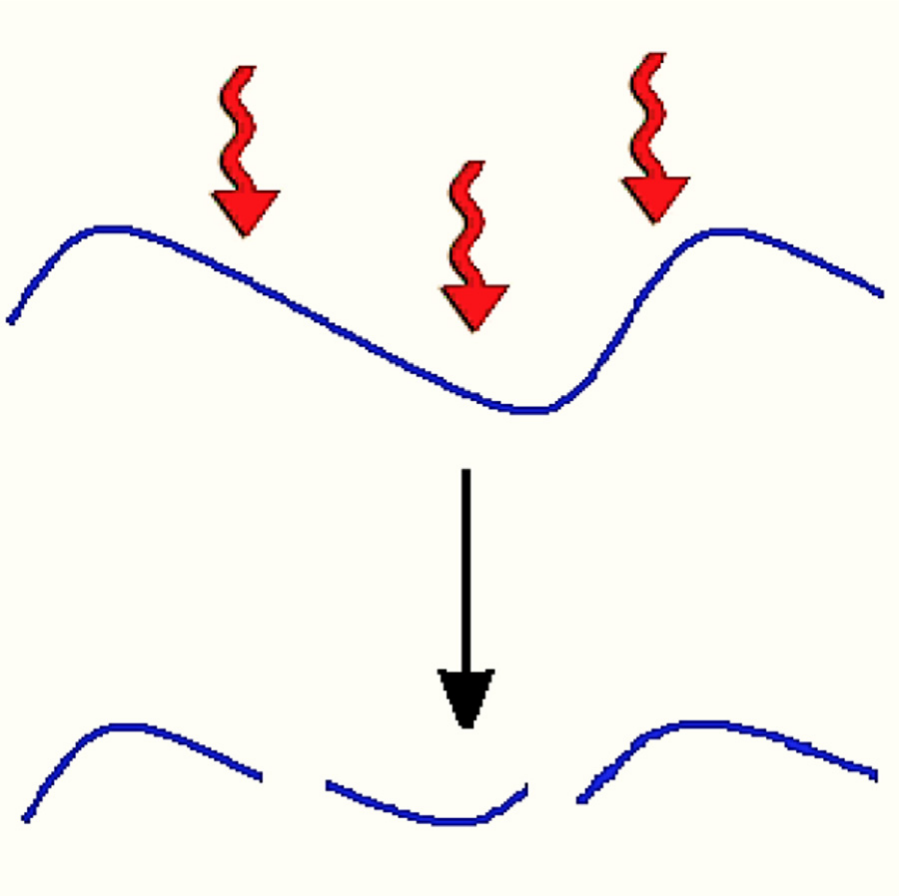
Chain degradation of bonds between glycoside groups, with each breakage adding one further chain to the total number of polymers in the system.

At any given time *t*, during the thermally activated degradation process, the state of the system can be represented by a the number of as yet unbroken bonds *N*(*t*) between the glycoside groups, and the total number of individual polymer chains at the same time, hereafter denoted as *n*(*t*). Since the number of glycoside groups that comprise a chain is always one more than the number of unbroken bonds in that same chain, then the total number of glycoside monomer residues in the system is *N*(t) + *n*(*t*). Furthermore, the degradation only breaks the bonds, but otherwise leaves the total number of monomers in the system unaltered. Thus, expressing this conservation of the number of glycoside in the process, one has:

(1) N(t)+n(t)=N(0)+n(0)

where, *N*(0) and *n*(0) are the initial number of unbroken bonds and that of chains in the system, respectively. The average molecular weight of the chains at time *t* is accordingly given by:

(2) $$M_{t}=m[N\left(t\right)+n\left(t\right)]/n\left(t\right)$$

where, *m* is the molecular weight of a single glycoside repeat unit. Now, it is also noted that each time a bond breaks one additional chain is generated, as is schematically displayed below in figure of Scheme 2.

Assuming then that the breakage of the glycoside bonds follows a first order kinetics, one can express the change in the number of broken bonds, and hence that of chains through following equation:

(3) $$-\frac{\mathrm{dn}}{\mathrm{dt}}=\frac{\mathrm{dN}}{\mathrm{dt}}=-\mathrm{kN}(t)$$

The constant *k* in the above equation is the rate constant for bond breakage, occurring as a result of thermal degradation. The rate constant will be a function of the nature and strength of bonds between the glycoside monomers, as well as the temperature of the system. Solving Eq. (3) for *N*(*t*) and substituting the resulting expression in Eq. (1), we arrive at the following relation for the time dependence variation of the number of chains in the system:

(4) $$n\left(t\right)=N\left(0\right)(1-e^{-kt})+n\left(0\right)$$

The average molecular weight of chains at time *t*, *M*_*t*_, is then:

(5) $$\frac{M_{t}}{m}=\frac{N\left(t\right)+n(t)}{n(t)}=\frac{N\left(0\right)+n\left(0\right)}{N\left(0\right)[1-\exp\left(-kt\right)]+n\left(0\right)}=\frac{\left(M_{0}/m\right)}{{(M}_{0}/m)-[N\left(0\right)/n\left(0\right)]\exp\;(-\mathrm{kt})}$$

where, *M*_0_ denotes the original average molecular weight of the polymer molecules, prior to the commencing of the degradation process. Now, recall that *N*(0)/*n*(0) is simply ${(M}_{0}/m)-1$ and by inverting both sides of the above equation, we arrive at the following result for the variation of the molecular weight of chains in our system with time:

(6) $$\frac{1}{M_{t}}=\frac{1}{m}\left[1-\exp\left(-\mathrm{kt}\right)\right]+\frac{1}{M_{0}}\exp\left(-kt\right)$$

It is seen that at very long times the above equation predicts that *M*_*t*_ = *m*. This is exactly the result expected, since at the end of the degradation process all bonds are broken and all chains are reduced to individual unconnected monomers. In the literature it is a common place for a semi-empirical relation of the following form to be used in order to describe the kinetics of degradation in polymers [3], [4]

(7) $$\frac{1}{M_{t}}=\frac{1}{M_{0}}+\frac{kt}{m}$$

It turns out that this equation can be derived from our Eq. (6), and represents the short time behavior of the degradation kinetics. Often in such work, involving a study of the loss of viscosity due to polysaccharide degradation, it is short time behavior that is of prime interest and this is all that is often measured. At longer times, the chains become too short and the drop in viscosity too extensive for it to be of any real consequence. Thus, Eq. (7) is a good approximation in most practical cases. To see how this equation arises from the more accurate result given here by Eq. (6), we note that at times *t* << 1/*k* the exponential terms in the latter equation can be expanded to first order in time. Then:

(8) $$\frac{1}{M_{t}}=\left(\frac{\mathrm{kt}}{m}\right)+\frac{1}{M_{0}}\left(1-\mathrm{kt}\right)=\left(\frac{\mathrm{kt}}{m}\right)(1+\frac{m}{M_{0}})+\frac{1}{M_{0}}$$

This can be further simplified to Eq. (7), given that almost in all cases of interest the initial molecular weight of chains is far larger than that of a single glycoside monomer, i.e. *M*_0_ >> *m*.

Experimental details for the thermally treated blend films are described in Ref. [1]. The κ-carrageenan solution used in this work prepared with dissolving 0.275 g of κ-carrageenan (the point of coil-overlap concentration) for obtaining 0.275 g/dL. Separately, the κ-carrageenan treated at the temperature of 80 °C at various time of 60–240 min, and then incorporated to chitosan solution for synthesizing a treated blend film.

#### 2.5.2 Data concerning effect of κ-carrageenan degradation on physical and optical properties of blend

By using a combination of acetic acid (2% (v/v)) and ascorbic acid (0.4 g/dL), providing a reduction in the otherwise strong affinity between κ-carrageenan and chitosan, a homogenous blend film with uniform thickness was fabricated. In comparison, the common acetic acid solvent technique, which is usually used for preparing a blend of chitosan film by a solvent casting method [1], [5], causes difficulties in fabricating a blend of chitosan film with anionic polymer. This is due to the fact that the cationic chitosan matrix creates a strong affinity with other anionic polymers, providing an aggregation-precipitation complex. Therefore, the chitosan cannot provide an even and uniform film with applying common solvent casting method.

Data here is for the physical and optical properties of the chitosan/κ-carrageenan blend film. The appearance of the neat chitosan film (with no plasticizer) was opaque, having a non-flexible and brittle structure. After blending of chitosan with untreated κ-carrageenan, the film was seen to be more flexible, displaying a smooth and uniform surface. The color of homogeneous intact blend film reflects the high level of uniformity of the film, albeit the same was not observed in the other treated blend films. The apparent color of neat chitosan changed from yellow to purple on addition of untreated κ-carrageenan, which could be easily observed by the naked eye. For more insight into the color and transparency see Table 1.

The measured Hunter parameters of L^*^, a^*^, and b^*^ for the neat chitosan film were 71.3, +3.6 and +14.5, respectively, where L^*^, a^*^, and b^*^ are the lightness, red-green coordinate and yellow-blue coordinate. The greater value of b^*^ was indicative of yellowish color for chitosan, as expected. The color parameters of the chitosan film were not affected by incorporation of untreated κ-carrageenan, excluding a* index. On the other hand, the color parameters of chitosan were considerably affected with incorporation of treated κ-carrageenan. A significant color change was observed for T_240_ sample, in which L^*^ index increased to 78.5, while b^*^ decreased to +6.2.

Transparency is one of the main physical properties of the packaging films, providing see-through character or prevents light transmission [6]. Table 1 also shows influences of introducing untreated and treated κ-carrageenan on the transparency of the chitosan-based film. The inclusion of untreated κ-carrageenan could not change the transparency value of the chitosan film. In the same way, the transparency of the chitosan/treated κ-carrageenan film did not change in comparison to the neat chitosan film. Nevertheless, in the case of T_240_, heating for 240 min was coincided with a significant reduction in turbidity.

The film thickness values are presented in Table 1. It was observed that the thickness of the neat chitosan film was very similar to the treated films, except for T_240_ case (*p*<0.05). The thickness of T_240_ was 45.4 µm, which was considerably less than the neat chitosan film with a value of 57.7 µm.

#### 2.5.3 Model dataset for fitting equilibrium moisture content

We have fitted three models include GAB, BET and Smith models to the data. The GAB model, which had high coefficient of determination *R*^2^≥0.98 with a low standard error (Table 2), was used to fit the equilibrium moisture content curve.

#### 2.5.4 Water contact angle observation dataset

**Fig. 6:**
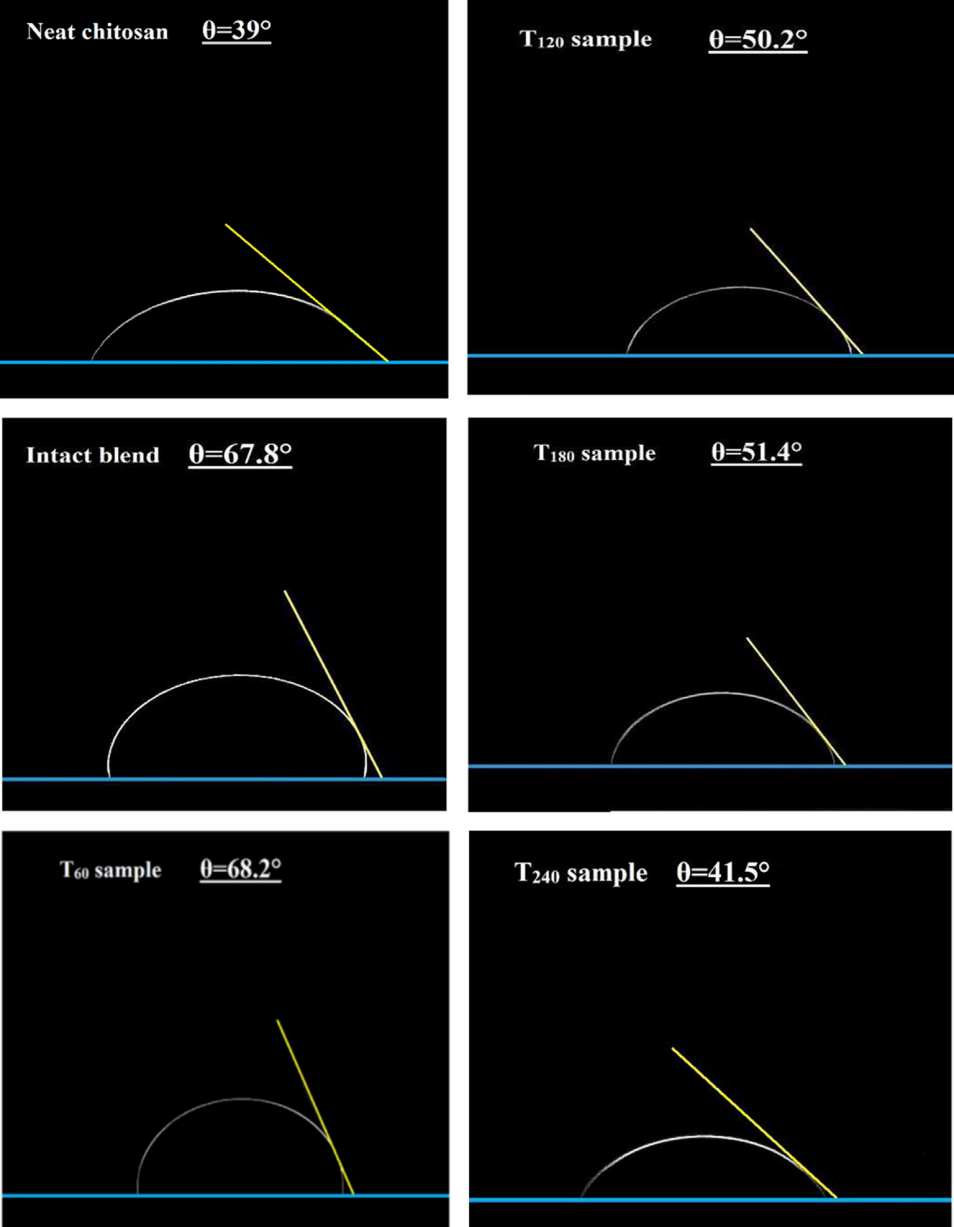
Contact angle images for various films, after 30 s following deposition, obtained at the ambient temperature.

The water contact angle images dataset of pure chitosan and its blend films are displayed in Fig. 6. The surface hydrophobicity of the pure chitosan film exhibits a low contact angle with a value of *θ*=39.0°, with water droplet fully absorbed into the film matrix after 60 s. The hydrophobicity image demonstrated that the incorporation of untreated κ-carrageenan increased the surface hydrophobicity of the chitosan film, demonstrated by a further increase of 28.8° in the value of the contact angle. A similar trend in surface hydrophobicity of T_60_ was observed, with its contact angle reaching a value of *θ*=68.2°. In contrast, the contact angle images show an apparent deterioration in the surface hydrophobicity of the thermally treated samples (T_120_–T_240_). The reduction in the surface hydrophobicity demonstrates the lack of sufficient hydrophilic sites on κ-carrageenan to interact with chitosan functional groups. This is also reflected in a reduced rigidity for the blend [7], [8]. The larger number of nonassociated chitosan functional groups in turn also increases the hydrophilicity of the film.

## Figures and Tables

**Table 1:** Physical and optical properties of various films. The samples were coded as T_60_, T_120_, T_180_ and T_240_, in which superscript implies the dissolution time of the κ-carrageenan at 60, 120, 180 and 240 min, respectively.

Film type	Thickness (µm)	WS (%)	Optical characteristic				
			L*	a*	b*	*ΔE*	Transparency (T_600_×μm^−1^)
Chitosan	57.7±0.7^a^	27.3±0.6^a^	71.3±0.8^a^	3.6 ±0.01^a^	14.5±0.08^a^	–	1.12 ± 0.02^a^
Intact blend	56.6±0.6^a^	23.3±1.1^b^	72.6±0.5^a^	4.6±0.04^b^	13.5±0.05^a^	1.92	1.10±0.01^a^
T_60_	56.7±0.7^a^	22.8±1.0^b^	71.1±0.2^a^	4.6±0.02^b^	13.9±0.04^a^	1.33	1.12 ± 0.01^a^
T_120_	57.4±1.1^a^	18.5±0.5^c^	73.0±0.5^a^	4.4±0.04^b^	13.3±0.06^a^	2.23	1.13 ± 0.02^a^
T_180_	58±0.4^a^	23.6±0.9^b^	75.7±0.9^b^	3.7±0.03^a^	11.4±0.06^b^	5.51	1.16 ± 1.1^ab^
T_240_	45.4±14^b^	26.7±0.7^a^	78.5±0.9^c^	2.4±0.04^c^	6.2±0.03^c^	11.01	1.19 ± 2.1^b^

**Table 2:** Estimated GAB, BET, Smith constants and monolayer moisture levels for various films, where *M*_0_ is monolayer moisture content in g H_2_O/g solid and *C*, *K*, *C*_1_ and *C*_2_ are constants.

**Film type**	**GAB**				**BET**			**Smith**		
	***M***_**0**_	***C***	***K***	***r***^**2**^	***M***_**0**_	***C***	***r***^**2**^	***C***_**1**_	***C***_**2**_	***r***^**2**^
Chitosan	0.082	1.13	0.85	0.98	2.23	0.082	0.96	−0.0047	−0.1396	0.97
Intact blend	0.56	0.29	0.94	0.99	2.24	0.088	0.98	0.0005	−0.1531	0.98
T_60_	0.53	0.32	0.92	0.99	2.57	0.011	0.98	0.0021	−0.1579	0.98
T_120_	1.22	0.18	0.95	0.99	3.51	0.068	0.97	0.0089	−0.1616	0.99
T_180_	1.73	0.12	0.98	0.99	3.06	0.074	0.98	0.011	−0.1578	0.98
T_240_	0.27	0.43	0.91	0.99	1.7	0.09	0.98	−0.0018	−0.1210	0.98
