# Supplementary material for: Physico-mechanical analysis data in support of compatibility of chitosan/κ-carrageenan polyelectrolyte films achieved by ascorbic acid, and the thermal degradation theory of κ-carrageenan influencing the properties of its blends
Source: Data Brief. 2016 Oct 1;9:648–60. doi: 10.1016/j.dib.2016.09.039 (PMC5067099; doi:10.1016/j.dib.2016.09.039)
Supplement: Supplementary file 1 — Supplementary material [file mmc1.docx]

**Statement on Conflict of interest**

To the best of our knowledge none of the authors has any direct or indirect financial or personal interest or belief that could affect their objectivity or in any way may inappropriately influence their actions. We confirm that no potential conflict of interest exists with regards to the publication of the work reported in the current article.

Yours faithfully

R. Ettelaie
